# Supplementary material for: Amanita Section Phalloideae Species in the Mediterranean Basin: Destroying Angels Reviewed
Source: Biology (Basel). 2022 May 18;11(5):770. doi: 10.3390/biology11050770 (PMC9138314; doi:10.3390/biology11050770)
Supplement: Supplementary file 1 [file biology-11-00770-s001.zip › Supplementary Table S2.pdf]

Table S2: Amatoxins and phallotoxins contents in *Amanita* species (mg/g dry weight)

	Collection date	α-Amanitin	β-Amanitin	Phallacidin	Phalloidin	Phallotoxins/ Amatoxins
A. virosa (Sweden)<br>PAM Su06-19, LIP:0402243	2012	3.34 ± 0.25	< 0.039	3.27 ± 0.28	1.35 ± 0.10	1.38
A. amerivirosa<br>PAM19110002, LIP:0401672	2019	< 0.039	0.11 ± 0.003	< 0.039	1.78 ± 0.01	15.21
A. amerivirosa<br>PAM19110001, LIP:0401671	2019	< 0.039	0.092 ± 0.013	< 0.039	2.55 ± 0.28	25.25
*A. verna**<br>PAM01042903, LIP:0402249	2001	0.87 ± 0.04	3.22 ± 0.24	1.63 ± 0.08	<0.039	0.40
A. verna Spain<br>PAM19060001, LIP:0402244	2019	5.47 ± 0.20	10.26 ± 0.59	4.69 ± 0.12	0.64 ± 0.03	0.34
A. phalloides var. *dunensis*<br>PAM19110516, LIP:0402239	2019	4.43 ± 0.21	4.14 ± 0.20	6.22 ± 0.17	1.56 ± 0.04	0.91
A. phalloides<br>SMM2018-10, LIP:0402268	2018	1.16 ± 0.06	3.90 ± 0.13	5.30 ± 0.15	0.97 ± 0.04	1.24
A. vidua<br>PAM19050401, LIP:0401591 (a)	2019	7.18 ± 0.54	7.55 ± 0.69	6.53 ± 0.45	1.94 ± 0.11	0.58
A. vidua<br>PAM19050401, LIP:0401591 (b)	2019	7.08 ± 0.44	8.50 ± 0.57	7.38 ± 0.53	1.91 ± 0.13	0.60
A. vidua<br>PAM19050401, LIP:0401591 (c)	2019	4.93 ± 0.27	5.02 ± 0.23	7.78 ± 0.48	1.38 ± 0.10	0.92
A. vidua<br>PAM19050401, LIP:0401591 (average)	2019	6.40 ± 1.27	7.02 ± 1.80	7.23 ± 0.64	1.74 ± 0.32	0.67
*A. vidua**<br>Kizlik 83, LIP :0002253	1987	0.27 ± 0.01	0.80 ± 0.02	7.23 ± 0.63	0.13 ± 0.01	6.88

* Probable toxin degradation with age
