# Supplementary figures and images for: Amanita Section Phalloideae Species in the Mediterranean Basin: Destroying Angels Reviewed
Source: Biology (Basel). 2022 May 18;11(5):770. doi: 10.3390/biology11050770 (PMC9138314; doi:10.3390/biology11050770)

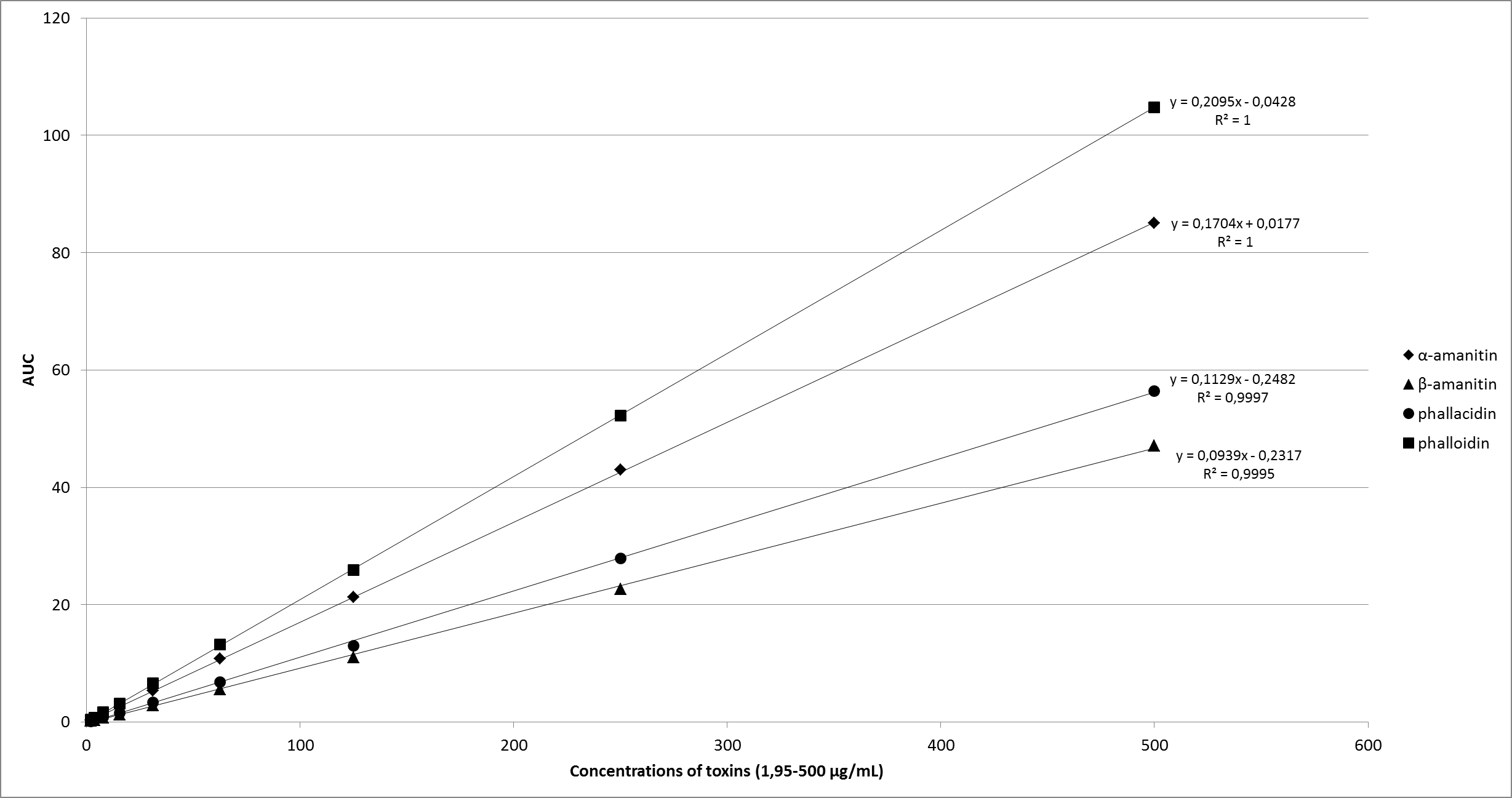

Supplement: Supplementary file 1 [file biology-11-00770-s001.zip › Supplementary Figure S1.png]
